# Clinical features and long-term surgical outcomes of conus medullaris hemangioblastomas

**DOI:** 10.1186/s41984-026-00548-4

**Published:** 2026-03-24

**Authors:** Liang Zhang, Bo Han, Wenqing Jia

**Affiliations:** 1 Department of Neurosurgery, National Cancer Center, National Clinical Research Center for Cancer/Cancer Hospital, Chinese Academy of Medical Sciences and Peking Union Medical College, Beijing, China; 2 Department of Neurosurgery, Beijing Tiantan Hospital, Capital Medical University, Fengtai District, Beijing, China; 3 Department of Neurosurgery, Beijing Tiantan Hospital, Capital Medical University, Beijing, China

**Keywords:** Conus medullaris, Hemangioblastoma, Von Hippel-Lindau, Intrameduallary, Microsurgery, Prognosis

## Abstract

**Background:**

Conus medullaris hemangioblastomas (HBs) are extremely rare vascular pathologies. The clinical-radiological features and long-term surgical outcomes of this rare disease are still lacking because of its rarity.

**Objective:**

This single-center study was carried out to describe the clinical and radiological characteristics and surgical outcomes of this rare entity.

**Methods:**

A cohort of patients who underwent resection of conus medullaris HBs in our center between 2009 and 2020 was retrospectively identified. Data on demographic features and surgical outcomes were collected and analyzed.

**Results:**

Fourteen patients were consecutively included in this study (mean onset age of 43.2 ± 14.6 years, 57.1% male). Ten (71.4%) of them were in the sporadic group, whereas four (28.6%) had von Hippel-Lindau (VHL) syndrome. Gross total resection (GTR) was achieved in most patients (*n* = 13, 92.9%), whereas subtotal resection (STR) was achieved in the remaining patient (7.1%). During the 96.9 ± 41.9 (range: 39–172) months of follow-up, six patients (42.8%) showed improved function, four patients (28.6%) remained the same, two patients (14.3%) worsened, and two patients (14.3%) died. No patients experienced lesion recurrence.

**Conclusion:**

Conus medullaris HBs are rare spinal vascular entities. Timely and careful microsurgical dissection of HBs can provide symptomatic relief and halt the progression of neurological deficits. The surgical outcome is usually satisfactory, even in recurrent cases.

**Supplementary Information:**

The online version contains supplementary material available at 10.1186/s41984-026-00548-4.

## Introduction

Spinal hemangioblastomas (HBs) are benign vascular-rich entities that can either arise with von Hippel-Lindau (VHL) syndrome or appear sporadically [1–5]. Intramedullary HBs are the third most common type of intramedullary tumor, followed by ependymomas and astrocytomas, and mostly affect the cervical and thoracic regions [1, 5, 6].

Conus medullaris HBs are rare intramedually HBs, with only 16 cases reported in previous literature [7–19]. The rarity of this vascular lesion precludes interpretation because of its nonspecific symptoms resulting from the mass effect on the conus medullaris. A delayed diagnosis may cause devastating damage to important neural elements of this region. Therefore, the current study was conducted to present the clinical-radiological features and long-term surgical results of patients in a single institution with HBs in the conus medullaris. A summary of previous reports of conus medullaris HBs was also conducted (Supplementary Table 1).

## Materials and methods

### Standard protocol approvals

This study was conducted after obtaining the consent of the Institutional Review Board (IRB) of our hospital. Due to the retrospective nature of this study, the requirement for informed consent was waived. All human studies were approved by the appropriate ethics committee and were performed according to the ethical standards set in the 1964 Declaration of Helsinki.

### Patients and data selection

Patients who were diagnosed with HBs in the conus medullaris after surgery from November 2009 to November 2020 were included in this study. Patients who experienced lesion recurrence were also included, and the duration of symptoms of these patients was calculated from the start of the recurrent symptoms or MRI confirmation of HBs to the second surgery. The demographic data (age, sex, presenting symptoms), magnetic resonance imaging (MRI) characteristics (lesion location, signals, maximum size, volume, flow void sign, and syrinx), extent of resection (EOR), and follow-up data (neurological function changes and recurrence) were electronically retrieved and analyzed.

### Management modality

The microsurgery was performed through the conventional posterior midline approach, with somatosensory evoked potentials (SSEP) and motor evoked potentials (MEP) monitoring of neurological function [6, 20–22]. An ultrasound was also used to detect HBs location and the surrounding tissues [23].

### Data definition

The size of the HBs was calculated as the largest diameter (antero-posterior, transverse, or craniocaudal dimension) on contrast-enhanced MR images, not including the surrounding edema and cyst/syrinx. Tumor volume was calculated via ellipsoid formula (transverse×anteroposterior×craniocaudal dimensions)/2. The modified McCormick scale (MMCS) was used to assess neurological function status (Table 1) [24]. The EOR was defined as gross total resection (GTR), subtotal resection (STR), or partial resection (PR). The EOR was evaluated by neurosurgeons and a senior board-certified neuroradiologist using contrast-enhanced MR images obtained within 3 days after surgery. The diagnosis of VHL syndrome was made by genetic analyses and clinical criteria (≧ 1 VHL syndrome-associated HBs with a family history of VHL syndrome or ≧ 2 VHL syndrome-associated HBs without considering family history) [5].

**Table 1:** Modified McCormick scale (MMCS) for evaluation of spinal neurological function

Grade	Definition
1	Neurologically intact, ambulates normally, may have minimal dysesthesia
2	Mild motor or sensory deficit, maintains functional independence
3	Moderate deficit, limitation of function, independent with external aid
4	Severe motor or sensory deficit, dependent with external aid
5	Paraplegia or quadriplegia

### Follow-up

After discharge, patients were followed at 3, 6 and 12 months at outpatient department, and subsequently assessed yearly by telephone or outpatient visits. MRI was performed at each visit in the first year and then for new onset of symptoms and widening the interval thereafter according to patients’ compliance or neurological function changes [6, 22]. The last follow-up was made in May 2024 either at the outpatient clinics or by telephone, and all patients were followed up.

### Statistical analysis

We compared features of VHL and sporadic group by using Student’s t-test (normally distributed) or Mann-Whitney U test (non-normal distribution) in continuous variables, and χ² or Fisher’s exact tests in categorical variables. All analyses were performed using IBM SPSS Statistics for Windows (version 25.0; IBM Corp., Armonk, NY, USA) with two-tailed *p* < 0.05 considered statistically significant.

## Results

### Baseline characteristics

Fourteen consecutive patients (8 men, 6 women) met the inclusion criteria. Mean age was 43.1 ± 14.6 (range: 14–65) years. Four individuals (28.6%) carried VHL disease, the remaining ten (71.4%) were sporadic cases. The mean duration of symptom was 11.7 ± 16.6 (range: 1–60) months, with VHL-positive subjects exhibiting a shorter duration of symptoms than sporadic cases (6 vs. 14 months, *p* = 0.042) (Table 2, Supplementary Table 2).

**Table 2:** Baseline characteristics of 14 patients with conus medullaris hemangioblastomas (HBs)

No.	Age (yrs)	Sex	Duration (mos)	Symptoms and signs	Site	VHL	Recurrent	Spinal HB	Resection	Bleeding (ml)	FU (mos)	MMCS		
												Pre	Post	FU
1	56	M	1	Pain+weakness	T12-L2	No	Yes	1	GTR	100	113	2	1	1
2	18	M	2	Pain+weakness+numbness+sphincter disorders	L1	Yes	No	2	GTR	100	172	2	2	2
3	56	F	2	Leg weakness+sphincter disorders	T12	Yes	No	3	GTR	150	43	3	3	-
4	44	F	12	Weakness+numbness+sphincter disorders	T12	No	Yes	1	GTR	300	103	3	5	4
5	46	F	36	Numbness	T12	No	Yes	1	GTR	50	106	1	1	1
6	62	M	3	Pain+weakness+numbness	T12	No	Yes	1	GTR	100	125	3	4	3
7	14	F	9	Pain+weakness	T12-L1	No	No	1	GTR	200	153	2	2	1
8	47	M	60	Weakness+numbness+sphincter disorders	T11-L2	No	No	1	STR	2000	90	3	4	4
9	39	M	1	Pain+sphincter disorders	T12-L1	No	No	1	GTR	100	98	2	2	1
10	65	M	12	Weakness+sphincter disorders	T12-L1	No	No	1	GTR	400	41	3	3	2
11	41	F	5	Pain+weakness	T12	No	No	1	GTR	100	39	2	2	1
12	41	F	12	Pain+numbness+weakness+sphincter disorders	T12	Yes	No	1	GTR	100	136	3	2	-
13	41	F	8	Pain+weakness+sphincter disorders	T12-L1	Yes	No	1	GTR	50	56	2	2	1
14	34	M	1	Pain	T12-L1	No	No	1	GTR	100	82	1	1	1

mos, months; yrs, years; F, female; M, male; GTR, gross total resection; STR, subtotal resection; FU, follow-up; VHL, von Hippel-Lindau; MMCS, Modified McCormick scale

Previous surgical resection of posterior-fossa HBs had been performed in two patients (medulla, *n* = 1; cerebellum, *n* = 1), and one had undergone Gamma-Knife radiosurgery for a cerebellar lesion. Multifocal spinal HBs was documented in two patients (cauda equina, *n* = 1; T10 and T11, *n* = 1). Intra-operative indocyanine green (ICG) videoangiography was employed in one patient (case 11), none had undergone pre-operative spinal angiography or embolization. Four patients (28.6%) who experienced lesion recurrence 64.3 ± 52.9 (range: 12–120) months after the first surgery were also included in this study. Their neurological status had deteriorated, justifying re-operation at our center.

At admission, the main symptom was leg weakness (*n* = 11, 78.6%), followed by radicular pain (*n* = 9, 64.3%), sphincter dysfunction (*n* = 8, 57.1%) and sensory deficits (*n* = 6, 42.9%). Functional baseline, graded by the MMCS, was favorable (MMCS ≦ 2) in eight patients (57.1%). Severe pre-operative disability (score ≥ 3) were patients with symptom duration ≥ 12 months (*n* = 3), recurrent lesions (*n* = 2), concurrent brain or multifocal spinal HBs (*n* = 2), or VHL syndrome (*n* = 2) (Table 2).

### MRI characteristics

**Fig. 1:**
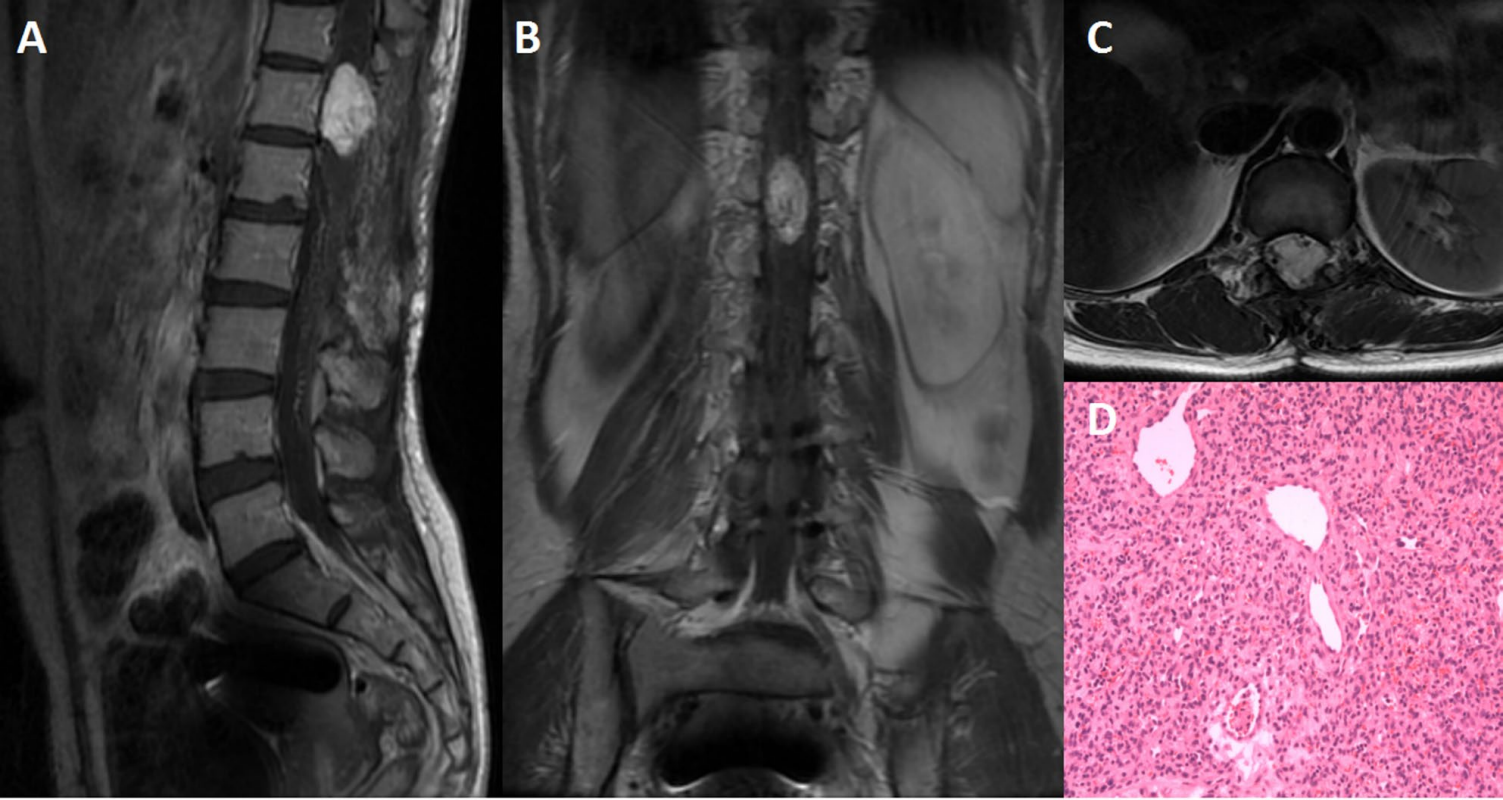
A 44-years old female patient had underwent surgery 10 years ago and suffered from worsening of leg weakness. MRI scans revealed a large solid intramedullary mass which was significantly enhanced on sagittal (**A**), and coronal (**B**) view. The lesion was hyperintense on axial T2WI (**C**), with vascular flow-void sign below the lesion. The flow-void sign could also been seen. The lesion was totally removed, and patholagically diagnosed as HBs, hematoxylin and eosin (H&E) stain, original magnification × 100 (**D**)

**Fig. 2:**
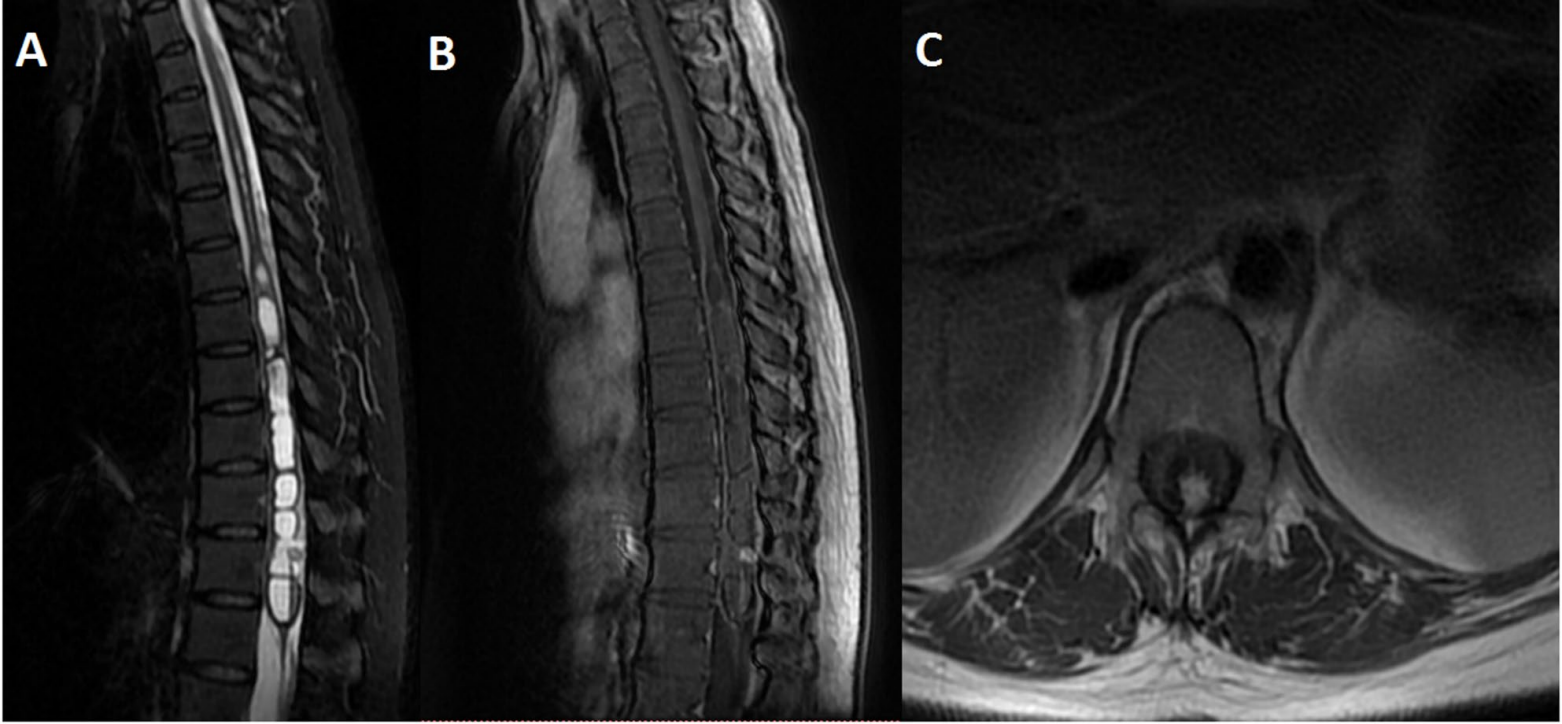
MRI showed a well-defined small intramedullary mass. The lesion appeared hypointense on sagittal T2WI (A) and axial T2WI (**C**), a nodular enhancement after gadolinium infusion (**B**). Syrinx could also be seen above and below the nodular (**A**)

The mean size and volume were 2.4 ± 2.7 (range: 0.5–11) cm and 2.8 ± 5.4 (range: 0.03–20.6) cm^3^, respectively. Sporadic HBs were larger than VHL-associated lesions in size (2.9 vs. 0.9 cm) and volume (3.8 vs. 0.3 cm^3^), though they were not statistical significant. (Table 3, Supplementary Table 2). On T1-weighted images (T1WI), 12 masses (85.7%) were iso-intense to spinal cord; seven (50%) were hyper-intense on T2-weighted images (T2WI), whereas all exhibited homogeneous enhancement after gadolinium. Syringomyelia was present in 13 cases (92.9%), and perilesional flow voids were observed in 12 (85.7%) (Figs. 1 and 2).

**Table 3:** Spinal MRI features and intraoperative findings of 14 patients with conus medullaris hemangioblastomas (HBs)

No	T1WI	T2WI	Enhancement	Size (cm)	Syrinx	Intraoperative findings	Texture
1	Iso	Hyper	Homo, obvious	4 × 2 × 1.5	No	Yellow, tight to spinal cord	Firm, solid
2	Iso	Hypo	Homo, obvious	1 × 1 × 1	Yes	High-vascular, obvious vessels	Firm, solid
3	Iso	Hyper	Homo, obvious	0.8 × 0.8 × 0.6	No	Reddish	Soft, solid
4	Iso	Hyper	Homo, obvious	3 × 2 × 1.5	Yes	Reddish, high-vascular, obvious vessels	Soft-firm, solid
5	Iso	Hyper	Homo, obvious	0.5 × 0.5 × 0.5	Yes	Reddish, capsule	Soft-firm, cystic-solid
6	Iso	Hyper	Homo, obvious	1 × 1 × 0.5	Yes	Reddish, obvious vessels	Soft-firm, solid
7	Iso	Iso	Hetero, obvious	3 × 1.3 × 1	Yes	Reddish, obvious vessels	Soft, solid
8	Iso	Iso	Homo, obvious	11 × 2.5 × 1.5	No	Reddish, tight to nerve root, high-vascular, obvious vessels	Firm, solid
9	Iso	Slight-hyper	Homo, obvious	1.5 × 1 × 1	No	Grey-reddish, high-vascular	Soft, solid
10	Hypo	Hypo	Homo, obvious	3 × 1 × 1	Yes	Reddish, obvious vessels	Soft, solid
11	Iso	Hypo	Homo, obvious	0.6 × 0.6 × 0.5	Yes	Reddish, capsule, obvious vessels	Soft, solid
12	Iso	Hypo	Homo, obvious	0.6 × 0.7 × 0.3	Yes	Reddish, high-vascular	Soft, solid
13	Hyper	Hypo	Homo, obvious	1 × 1 × 0.5	Yes	Reddish, high-vascular, obvious vessels	Soft, solid
14	Iso	Hyper	Homo, obvious	2 × 2 × 1.5	Yes	Yellow, tight to spinal cord and nerve	Firm, solid

T1WI, T1 weighted image; T2WI, T2 weighted image; Iso, isointense; hyper, hyperintense; hypo, hypointense; hetero, heterogeneous; homo, homogeneous

### Intraoperative findings

The operation was performed via standard posterior mid-line approach, laminectomy was extended to fully expose the hyper-vascular nodule. Microsurgical arachnoid dissection established a clear gliotic plane. Tiny arterial feeders were coagulated and divided in a circumferential fashion, the HBs were then coalgulated and removed in an en bloc fashion, whereas the dominant draining vein was not coalgulated until the final stage. The tumor cavity was inspected carefully.

One large HB (7.1%) extended from T11-L2 and adhered tightly to the conus medullaris and several abnormal vessels without a clear border. The surgical process was challenging, the blood loss volume was 2000 ml, and total removal was deemed unsafe and unachievable (Case 8). He received intra-operative blood transfusions for extensive intraoperative bleeding. For the remaining 13 HBs (92.9%), GTR was verified by post-operative MRI within 48 h. Mean operative blood loss was 309 ± 571 (range: 50 − 2 000) ml. Histopathology confirmed World Health Organization (WHO) grade 1 HBs in every specimen.

### Surgical outcomes and follow-up data

Neurological deterioration manifested as leg weakness occurred in four patients (28.6%); three concurrently experienced sensory disturbance. Leg weakness improved, but sensory problems did not improve by discharge. No patient developed cerebrospinal fluid (CSF) leakage, wound infection or fever.

During a mean follow-up of 96.9 ± 41.9 (range: 39–172) months, two patients (14.3%) died from unrelated causes (lung cancer, case 12; COVID-19 pneumonia, case 3). Both deceased patients were clinically evaluated prior to their unrelated deaths. One patient achieved functional improvement and one remained stable. Symptom-specific outcomes varied: Case 12 demonstrated improvement in pain, numbness, and leg weakness, whereas Case 3 showed no improvement in leg weakness or sphincter function. Among the 12 surviving patients, functional status improved in six (50%), remained stable in four (33.3%) and deteriorated in two (16.7%). Pain, leg weakness, numbness, and sphincter dysfunction improved in 87.5% (7/8), 77.8% (7/9), 60% (3/5), and 33.3% (2/6), respectively. No radiological recurrence was observed after GTR; the single residual lesion in Case 8 remained stable throughout 90 months of surveillance.

## Discussion

This study was conducted to summarize the clinical-radiological features and long-term surgical outcomes of patients with conus medullaris HBs in a single center. We found that total surgical resection could provide satisfactory outcomes and should be recommended in a timely manner, and repeated microsurgery can also benefit patients with recurrent lesions.

### Demographic characteristics

The mean onset age was 43 years in our cohort, which corresponded with that of patients with spinal HBs or cauda equina HBs [6, 22]. To date, only 16 cases of HBs in the conus medullaris have been reported, which was shown in supplementary Table 1 [7–19]. We encountered two children (≦ 18 years old) with sporadic conus medullaris HBs, which has reported only in one case before. The slight male predominance in this study was in accordance with previous reports [18].

Patients usually present with multiple manifestations (*n* = 12, 85.7%) of weakness, pain, sphincter disorders or numbness. These symptoms were mainly due to the space-occupying effect of the HBs and the accompanying syrinx or edema to the conus medullaris. The duration of symptoms varied and was shorter in the VHL group than in the sporadic group (6 vs. 14 months). The various symptom types and duration might suggest that the clinical behavior was different between the sporadic and VHL groups.

### MRI findings

HBs usually feature isointensity on T1WI, hyperintensity on T2WI, and obvious and homogeneous enhancement on spinal MRI. However, these MRI features are not specific to HBs [6, 12]. HBs of the conus medullaris also feature edema or an associated syrinx as lesions in the cervical and thoracic regions [15, 25, 26]. Most lesions featured vascular flow void signs and an associated syrinx around the HBs in this cohort. These characteristics may be helpful for correctly diagnosing HBs in conus medullaris. The correct diagnosis is essential for planning a surgical strategy that can help minimize hemorrhage due to the high vascularity of HBs and minimize manipulation of the conus medullaris.

### Treatment modalities and prognosis

Total surgical resection is the optimal treatment modality, which can relieve the mass effect and prevent further damage to the conus medullaris. However, the surgical process is highly challenging because of not only the high vascularity of HBs but also the complex neurostructures in these locations. Manipulating lesions in this region may cause neurological deterioration, such as limb weakness, sensory disorders or sphincter disturbance. A previous study revealed that patients experienced worsening of neurogenic bladder and new paraparesis [18]. As demonstrated in our cohort, four patients (28.6%) experienced transient worsening of leg weakness, and three (21.4%) also experienced sensory problems. One patient (7.1%) with large HBs experienced massive bleeding due to its high vascular nature. We suggest not only maximal surgical removal of HBs but also preservation of neurological function.

The long-term surgical outcomes of conus medullaris HBs are unclear because of the rarity and short-term follow-up in previous reports [16, 18]. We included 4 patients who experienced lesion recurrence at 12–120 months, although GTR was achieved at the first surgery. A repeated surgery in our center was introduced after careful evaluation, and their preoperative function improved in approximately half of them at the long-term follow-up. No recurrence in this cohort was observed after surgery in our center. Further long-term follow-up is still needed in these patients.

### Limitations of study

This is a retrospective study and potential biases of this kind of study exist due to the limited sample size. However, this is still the largest single center case series of conus medullaris HBs reported to date.

## Conclusion

The diagnosis of HBs should be considered when an enhanced nodule in the conus medullaris with an associated syrinx and vascular flow void is present. Total timely and careful surgical resection can prevent neurological deficits and can lead to satisfactory outcomes, even for recurrent lesions. A life-long follow-up is needed considering the potential for recurrence, especially for VHL patients.

## Supplementary Information

Supplementary Material 1

## Data Availability

Not applicable.

## Abbreviations

HBs: hemangioblastomas
VHL: Von Hippel-Lindau
GTR: Gross total resection
STR: Subtotal resection
IRB: Institutional Review Board
MRI: Magnetic resonance imaging
EOR: Extent of resection
SSEP: Somatosensory evoked potentials
MEP: Motor evoked potentials
MMCS: Modified McCormick scale
PR: Partial resection
ICG: Indocyanine green
T1WI: T1-weighted images
T2WI: T2-weighted images
WHO: World Health Organization
CSF: Cerebrospinal fluid
H&E: Hematoxylin and eosin
