# Supplementary material for: Clinical features and long-term surgical outcomes of conus medullaris hemangioblastomas
Source: Egypt J Neurosurg. 2026 Mar 24;41(1):35. doi: 10.1186/s41984-026-00548-4 (PMC13013191; doi:10.1186/s41984-026-00548-4)
Supplement: Supplementary file 1 — Supplementary Material 1 [file 41984_2026_548_MOESM1_ESM.docx]

Supplementary table 1: Summary of conus medullaris hemangioblastoma in previous reports

| Author & Year | Age (years) | Sex | Location | VHL | Preoperative symptoms | Duration of symptoms (mos) | Size (cm) | Duration of symptoms (mos) | Syrinx | EOR | Recurrence | FU (mos) | Outcomes |
| --- | --- | --- | --- | --- | --- | --- | --- | --- | --- | --- | --- | --- | --- |
| [Michaud et al. 1988](https://pmc.ncbi.nlm.nih.gov/articles/PMC7806181/#R7) | 0.5 | F | T12-L1 | No | No | – | – | – | – | – | No | 6 | No new deficits |
| [Spetzger et al. 1996](https://pmc.ncbi.nlm.nih.gov/articles/PMC7806181/#R10) | 44 | F | T11-L2 | – | Severe paraparesis, genito-sphincteric disturbances |  |  |  | No |  |  |  | Improved |
|  | 65 | M | T12-L1 | – | Ataxia, moderate genito-sphincteric disturbances | – | – | – | Yes | – | – | – | Improved |
| [Chu et al. 2001](https://pmc.ncbi.nlm.nih.gov/articles/PMC7806181/#R8) | 30 | M | T12-L1 | Yes | Paraparesis,hypesthesia, sense decreased, sphincteric disturbances | 60 | 6 | 60 | Yes | – | – | – | – |
| [Malis L. 2002](https://pmc.ncbi.nlm.nih.gov/articles/PMC7806181/#R14) | 21 | F | T12 | No | Low-back pain, leg weakness, numbness and dysesthesia | – | 2.1*1.5 | – | Yes | Totally | No | 168 | Improved |
| Roonprapunt et al. 2003 | 31 | F | T12-L1 | No | Pain, paresthesias, urinary incontinence | 8 | 14 | 8 | – | – | No | 20 | Unchanged |
| [Lee et al. 2003](https://pmc.ncbi.nlm.nih.gov/articles/PMC7806181/#R3) | 27 | F | T11-L1 | No | Low back pain, leg paresthesia, urinary difficulty | 5 | 2 | 5 | Yes | Totally | – | 39 | Unchanged |
| [Van-Velthoven et al. 2003](https://pmc.ncbi.nlm.nih.gov/articles/PMC7806181/#R6) | 27 | F | T12 | Yes | Leg numbness | 8 | – | 8 | – | – | No | 27 | Improved |
| [Biondi et al. 2005](https://pmc.ncbi.nlm.nih.gov/articles/PMC7806181/#R11) | 24 | F | T12-L1 | No | – | – | – | – | Yes | – | No | – | Improved |
| [Chen et al. 2008](https://pmc.ncbi.nlm.nih.gov/articles/PMC7806181/#R5) | 74 | M | T12-L1 | – | Leg weakness, neurological bladder | – | – | – | Yes | partially removed | – | – | Improved |
| [Han et al. 2008](https://pmc.ncbi.nlm.nih.gov/articles/PMC7806181/#R12) | 57 | M | L1-L2 | – | Low back pain | 9 | – | 9 | – | Totally | No | 81 | Unchanged |
|  | 61 | M | T12-L1 | – | Pain, paresthesias, weakness | 1 | – | 1 | – | subtotally | No | 23 | Unchanged |
|  | 37 | F | T12-L1 | – | Pain, paresthesias, weakness | 38 | – | 38 | – | Totally | No | 13 | Worse (neurogenic bladder) |
| [Bostrom et al. 2008](https://pmc.ncbi.nlm.nih.gov/articles/PMC7806181/#R13) | – | – | – | – | – | – | – | – | – | – | – | – | Improved |
| Alvarez et al.2021 | 44 | M | T12-L1 | Yes | leg weakness, gait imbalance, absent perineal genital sensation | – | – | – | Yes(holocord) | Totally | – | – | Improved |
| Lee et al. 2023 | 30 | F | T12-L1 | Yes | Paresthesia | 5 | – | 5 | Yes | Totally | No | 6 | Worse (new hypoesthesia and aching sensations) |

Abbreviations: F, female; M, male; VHL, von Hippel-Lindau; mos, months; FU, follow-up; EOR, extent of resection.

Supplementary table 2: Comparison of baseline characteristics between sporadic and VHL-associated conus medullaris HBs

| Variable | Total, N=14 (%) | Sporadic, N=10 (%) | VHL, N=4 (%) | *P* |
| --- | --- | --- | --- | --- |
| Age (years)* | 43.1 ± 14.6 (14-65) | 44.8 ± 14.7 (14-65) | 39.0 ± 15.7 (18-56) | 0.551 |
| Sex |  |  |  |  |
| Male | 7 (50.0) | 6 (60.0) | 1 (25.0) | 0.559 |
| Female | 7 (50.0) | 4 (40.0) | 3 (75.0) |  |
| Symptoms |  |  |  |  |
| Motor | 11 (78.6) | 7 (70.0) | 4 (100.0) | 0.505 |
| Pain | 9 (64.3) | 7 (70.0) | 2 (50.0) | 0.580 |
| Sphincter disorders | 8 (57.1) | 5 (50.0) | 3 (75.0) | 0.580 |
| Sensory | 6 (42.9) | 4 (40.0) | 2 (50.0) | 1.000 |
| Symptoms ≧3 | 5 (35.7) | 3 (30.0) | 2 (50.0) | 0.580 |
| Duration of symptoms (mos)* | 11.7 ± 16.6 (1-60) | 14.0 ± 19.3 (1-60) | 6.0 ± 4.9 (2-12) | 0.042 |
| Pre-op MMCS≦2 | 8 (57.1) | 6 (60.0) | 2 (50.0) | 1.000 |
| Synchronous brain HBs | 2 (14.3) | 0 (0) | 2 (50.0) | 0.066 |
| Maximum size (cm)* | 2.4 ± 2.7 (0.5-11) | 2.9 ± 3.1 (0.5-11) | 0.9 ± 0.2 (0.7-1) | 0.208 |
| Maximum volume (cm^3^)* | 2.8 ± 5.4 (0.025-20.6) | 3.8 ± 6.2 (0.025-20.6) | 0.3 ± 0.2 (0.063-0.5) | 0.281 |

Abbreviations: *mos*, months; *MMCS*, modified McCormick scale; *VHL*, von Hippel-Lindau; *op*, operation; *HBs,* hemangioblastomas.

* Values are presented as mean ± standard deviation (range).

Bold means statistically significant differences.
